# Psychometric Properties of Multi-Dimensional Scale of Perceived Social Support in Chinese Parents of Children with Cerebral Palsy

**DOI:** 10.3389/fpsyg.2017.02020

**Published:** 2017-11-21

**Authors:** Yongli Wang, Qin Wan, Zhaoming Huang, Li Huang, Feng Kong

**Affiliations:** ^1^Education and Rehabilitation Department, Faculty of Education, East China Normal University, Shanghai, China; ^2^Rehabilitation Department, Yueyang Hospital of Integrated Traditional Chinese and Western Medicine, Shanghai University of Traditional Chinese Medicine, Shanghai, China; ^3^School of Psychology, Shanxi Normal University, Xi’an, China

**Keywords:** social support, confirmatory factor analysis, validity, reliability, measurement invariance

## Abstract

The Multi-dimensional Scale of Perceived Social Support (MSPSS) is one of the most extensively used instruments to assess social support. The purpose of this research was to test the reliability, factorial validity, concurrent validity and measurement invariance across gender groups of the MSPSS in Chinese parents of children with cerebral palsy. A total of 487 participants aged 21–55 years were recruited to complete the Chinese MSPSS and Parenting Stress Index-Short Form (PSI-SF). Composite reliability was calculated as the internal consistency of the Chinese MSPSS and a (multi-group) confirmatory factor analysis (CFA) was conducted to test the factorial validity and measurement invariance across gender. And Pearson correlations were calculated to test the relationships between MSPSS and PSI-SF. The Chinese MSPSS had satisfactory internal reliability with composite reliability values of more than 0.7. The CFA indicated that the original three-factor model was replicated in this specific population. Importantly, the results of the multi-group CFA demonstrated that configural, metric, and scalar invariance across gender groups was supported. In addition, all the three subscales of MSPSS were significant related with PSI-SF. These findings suggest that the Chinese MSPSS is a reliable and valid tool for assessing social support and can generally be utilized across sex in the parents of children with cerebral palsy.

## Introduction

Cerebral palsy (CP), as a common cause of disability in children, is a serious threat to parents’ psychological and physical health (Cheshire et al., 2010; Whittingham et al., 2013). Social support is an important resource to cope with various stressors (Campbell et al., 2011), and can mediate the influence of parenting stress on psychological and physical health in parents of children with CP (Jeong et al., 2013). In this study, we sought to validate a social support scale in a sample of Chinese parents of children with CP, because specific population groups may have different social support expectations, social networks, and cultural backgrounds than the normal adolescents, verification of the reliability and validity of assessment scales in different special groups is very necessary and useful (Kim et al., 2008).

Social support is a multidimensional construct, and the types of social support vary widely (Stanley et al., 1998). In numerous social support instruments, Multi-dimensional Scale of Perceived Social Support (MSPSS) is a considered ideal assessment tool (Bruwer et al., 2008). First, the MSPSS assesses different sources of support (i.e., family, friends, and significant others). Second, this instrument is brief yet comprehensive, easy to understand. The MSPSS is a 12-item questionnaire, which is a self-administered measure of perceived social support developed by Zimet et al. (1988). These authors found that the MSPSS had good reliability (with a Cronbach’s alpha of 0.85 to 0.91). Furthermore, confirmatory factor analysis (CFA) showed a robust three-factor structure (family support, friend support, and significant-other support) of the MSPSS existed (Zimet et al., 1988). In addition, the MSPSS was found to be negatively related to parenting stress, loneliness and depression, and positively related to self-esteem and life satisfaction, indicating good concurrent validity of the MSPSS (Dunst et al., 1986; Bruwer et al., 2008; Jeong et al., 2013; Zhao et al., 2013; Kong et al., 2015).

Over the years, the MSPSS has been translated into many other languages such as Thai, Malaysian, Swedish, Polish, and demonstrated good reliability and validity (Duru, 2007; Ng et al., 2010; Wongpakaran and Wongpakaran, 2012; Adamczyk, 2013; Ekbäck et al., 2013). In addition, adequate psychometric properties of the MSPSS are also found in some special groups such as psychiatric patients, implantable cardioverter defibrillator patients, patients with heart failure (Cecil et al., 1995; Pedersen et al., 2009; Shumaker et al., 2017). All these results suggested that the MSPSS possessed good psychometric properties.

The Chinese MSPSS was first established by the Hong Kong scholar Chou (2000), and later the traditional Chinese version was translated into simplified Chinese language by Guan et al. (2015). These two studies confirmed the Chinese MSPSS had good reliability and validity. However, they only used adolescents and college students as a sample. In addition, the reliability and validity of the Chinese MSPSS have also been verified in patients with methadone maintenance treatment (Zhou et al., 2015). In recent years, the worldwide incidence of CP is still around 0.2% (Oliveira and Matsukura, 2013; Nelson and Blair, 2015). CP children’s disorders in the development of posture and movement made the parents often experience higher levels of parenting stress (Ong et al., 1998), more chronic sorrow symptoms (Whittingham et al., 2013), and more irritable and anxious (Cheshire et al., 2010) than parents of normally developed children. Fortunately, there is evidence that social support has buffering effects on stressful life events and depression (Komatsu et al., 2010). Therefore, parents of children with CP need more social support to cope with stressful situations. However, parents of children with CP perceive low levels of social support than parents of children with normal development (Carona et al., 2013), parents of children with mentally retarded (Ashok, 2017), and rehabilitation professionals (Oliveira and Matsukura, 2013). There is no doubt that the social support of parents of children with CP is a matter of concern to researchers. A validated social support measurement tool for this particular group is a prerequisite for research. However, to our knowledge, the Chinese MSPSS has not been validated for the particular group of parents of children with CP. One purpose of this research was to examine the reliability, factorial validity and concurrent validity in relation to parenting stress of the Chinese MSPSS in parents of children with CP.

Another critical question when using the MSPSS is the measurement invariance across different gender groups. Prior studies have explored the difference in the MSPSS scores between fathers and mothers. For example, Ghorbani et al. (2014) found that the MSPSS scores of mothers of children with disabilities were lower than that of fathers. However, Simons et al. (2007) showed that there was no significant gender difference in the MSPSS scores. There may be a number of factors contributing to these differences, but before we discuss gender differences, we must first ensure that the MSPSS can measure the same latent factors across gender groups. Thus, another purpose of this research was to determine if the measurement structure underlying social support is equivalent across gender.

To address these issues, we firstly performed a CFA to test if the three-factor structure of the MSPSS can be replicated in Chinese parents of children with CP (*N* = 487). Secondly, we tested measurement invariance of MSPSS across gender groups by a multi-group CFA. Finally, we tested the concurrent validity of the Chinese version of the MSPSS in relation to parenting stress, which was selected due to their reliable negative relationship with social support (Jeong et al., 2013). We expected that perceived support from family, friends, and significant others will be inversely related to parenting stress.

## Materials and Methods

### Participants and Procedure

We used the MSPSS and PSI-SF to measure social support and parenting stress respectively. The questionnaires were mailed to directors of medical institutes, children’s rehabilitation centers, and special education schools in mainland China. The directors were responsible for sending to the parents, and the inclusion criteria were as follows: (1) the child’s age was between 1 and 12 years and has been diagnosed with CP; (2) the parents were Chinese-speaking and could independently complete the questionnaires. All participants signed a written informed consent and were asked to complete a set of questionnaires anonymously and voluntarily. Only one parent in a same family was asked to fill in the questionnaires. In total, we distributed 520 questionnaires and received 487 (mother = 366, father = 121) valid questionnaires for a recovery rate of 93.7%. The mean age of the participated parents is 33.69 (standard deviation = 6.12), age range is 21–55 years. The current study was approved by the Research Ethics Board of the local university.

### Measures

To measure social support, we used the Chinese version of the MSPSS. There are four items for each subscale of the Chinese MSPSS. A seven-point Likert scale is used (1 = very strongly disagree; 7 = very strongly agree). The complete scale ranges from 12 to 84. A higher score equates with higher social support. In this study, the Cronbach’s alpha coefficients of the family support (Items 3, 4, 8, 11), friend support (Items 6, 7, 9, 12), and significant-other support (Items 1, 2, 5, 10) subscales were 0.88, 0.89, and 0.87, respectively.

To measure parenting stress, we used the Chinese version of the Parenting Stress Index-Short Form (PSI-SF). The PSI-SF is a 36-item questionnaire developed by Abidin and Wilfong (1989) to measure parenting stress. It includes three factors: parental distress (PD) (Items 1–12), parent–child dysfunctional interaction (PCDI) (Items 13–23, 34), and difficult children (DC) (Items 24–33, 35–36). The form applies a five-point Likert scale (1 = strongly disagree; 5 = strongly agree). A higher score equates with higher parenting stress. The Chinese version of the PSI-SF has good reliability and validity (Yeh et al., 2001). In this study, the Cronbach’s alpha coefficients of the PSI-SF’s three factors were 0.90, 0.86, and 0.84, respectively.

### Data Analysis

To test if the three-factor structure of the MSPSS can be replicated in Chinese parents of children with CP, a CFA was firstly performed using Amos 23.0. Because the Chi-Square is considered very sensitive to the sample size, the following indices were used to evaluate model fit: comparative fit index (CFI), non-normal fit index (NNFI, also known as Tucker–Lewis Index), root mean square error of approximation (RMSEA), and standardized root mean square residual (SRMR) (Hu and Bentler, 1999). Accordingly, an acceptable model fit is indicated by a CFI of >0.90, a NNFI of >0.90, a RMSEA of <0.08, and a SRMR of <0.08. Values of CFI and NNFI above 0.95 (Hoyle, 1995), RMSEA and SRMR < 0.06 (Maccallum et al., 1996) represent an excellent model fit.

Then, the configural, metric, and scalar invariance were selected to examine the measurement invariance of Chinese MSPSS across gender groups by a multi-group CFA. The difference for CFI and RMSEA (ΔCF and ΔRMSEA) were used as indices for tests of invariance. ΔCF and ΔRMSEA of equal to or <0.01 were considered strong invariance (Cheung and Rensvold, 2002; Kong, 2017).

In addition, the relationships among perceived social support from family, friends, and significant others with parenting stress were examined by Pearson correlations. And *p* < 0.05 was considered statistically significant.

## Results

### Confirmatory Factor Analysis

**FIGURE 1:**
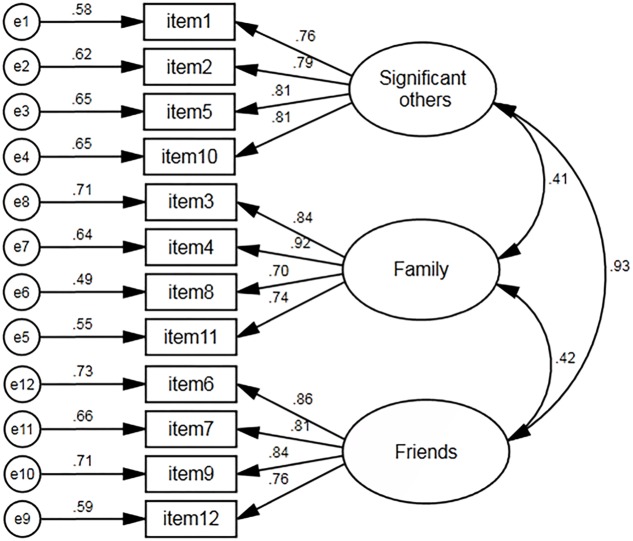
The structure model of Chinese MSPSS.

The CFA analysis found that all the fit indices of the three-factor model meet their corresponding criteria very well, χ^2^(51) = 170.67, *p* < 0.001, CFI = 0.969, NNFI = 0.959, RMSEA = 0.069, SRMR = 0.037. The factor loadings ranged from 0.70 to 0.92. The model of structure of social support was showed in **Figure 1**.

### Measurement Invariance

#### Configural Invariance

Configural invariance was first conducted to assess if the Chinese MSPSS has a three-factor structure for the gender groups. The result showed that the configural invariance model (M1) fitted the data very well, CFI = 0.967, RMSEA = 0.051 (**Table 1**), and all factor loading were significant (*p* < 0.05).

**Table 1:** Fit indices for measurement invariance across gender.

Model	χ^2^	df	CFI	NNFI	RMSEA	SRMR	Comparison	ΔCFI	ΔRMSEA
M1	229.42	102	0.967	0.957	0.051	0.052			
M2	224.13	111	0.965	0.959	0.050	0.047	M2 vs. M1	0.002	0.001
M3	267.68	123	0.962	0.959	0.049	0.048	M3 vs. M2	0.003	0.001


*CFI, comparative fit index; NNFI, non-normal fit index; RMSEA, root mean square error of approximation; SRMR, standardized root mean square residual; M1, configural invariance model; M2, metric invariance model; M3, scalar invariance model*.

#### Metric Invariance

Next, a metric invariance model (M2) was conducted to verify the invariance of factor loadings across the two groups. The results showed a good fit, CFI = 0.965, RMSEA = 0.050 (**Table 1**). And no significant changes occurred when compared to the M1, ΔCFI = 0.002, ΔRMSEA = 0.001.

#### Scalar Invariance

Scalar invariance implies invariance of item intercepts across groups. Finally, scalar invariance model (M3) was established to test the intercepts and factor loadings were constrained to be equal across the fathers and mothers, and the residual variances were freely estimated. The results showed the model fitted well, CFI = 0.962, RMSEA = 0.049 (**Table 1**). And no significant changes occurred when compared to the M2, ΔCFI = 0.003, ΔRMSEA = 0.001.

Taking these results together, it is suggested that configural, metric, and scalar invariance hold across gender groups.

### Reliability

Composite reliability was calculated as the internal consistency (Raykov, 1998) of the Chinese MSPSS. Compared to Cronbach’s alpha reliability, it does not assume that all indicators are equally reliable. If the composite reliability is more than 0.7, the model is good (Nunally, 1978). For all the parents, the composite reliability of the family support, friends support, and significant-other support subscales was 0.87, 0.88, and 0.89, respectively. For the mothers, the composite reliability of these three subscales was 0.86, 0.88, and 0.89, respectively. For the fathers, the composite reliability of these three subscales was 0.90, 0.88, and 0.90, respectively. These results indicated that the internal consistency of the Chinese MSPSS was good.

### Descriptive Analysis and Concurrent Validity

Concurrent validity is a supporting piece of evidence for construct validity. Pearson correlations were conducted with the total sample to test the relationship among perceived social support from family, friends, and significant others with parenting stress to assess the concurrent validity between the Chinese version of MSPSS and PSI-SF subscales. The result showed that MSPSS was significant related with PSI-SF. The mean and standardized deviations of variables were presented in **Table 2**.

**Table 2:** Mean, SD, and correlation between the variables.

	*M*	*SD*	1	2	3	4	5	6	7
(1) Family	20.4	6.11	1						
(2) Friends	17.1	6.23	0.515	1					
(3) Significant-other	16.9	6.50	0.492	0.841	1				
(4) Total MSPSS	54.4	16.27	0.769	0.912	0.906	1			
(5) PD	37.7	9.99	-0.325	-0.318	-0.261	-0.348	1		
(6) PCDI	29.2	8.25	-0.179	-0.212	-0.198	-0.227	0.526	1	
(7) DC	33.6	8.47	-0.156	-0.167	-0.173	-0.192	0.443	0.691	1
(8) Total PSI	100.4	22.31	-0.271	-0.284	-0.256	-0.313	0.810	0.868	0.833


*All correlations were significant at level of 0.01. The numbers 1–7 of the first line of the table represents the same content as the vertical numbers*.

In addition, we examined the relationship between age factors and social support, and found that the correlation between children’s age and social support was not significant (family: *r* = –0.011, *p* = 0.811; friends: *r* = –0.070, *p* = 0.124; significant-others: *r* = –0.068, *p* = 0.135; total MSPSS: *r* = –0.059, *p* = 0.191), and the correlation between parents’ age and social support was not significant (family: *r* = 0.027, *p* = 0.563; friends: *r* = –0.018, *p* = 0.697; significant-others: *r* = –0.087, *p* = 0.063; total MSPSS: *r* = –0.033, *p* = 0.483).

## Discussion

The present study aimed to investigate the reliability, factorial validity, measurement invariance across gender of the Chinese MSPSS in parents of children with CP. As we expected, the result showed that the three-factor structure was replicated in parents of children with CP. This is consistent with the original study by Zimet et al. (1988). In addition, the Chinese MSPSS had good internal consistency reliability with composite reliability values of more than 0.7. These results suggested that the Chinese MSPSS is a reliable and valid measure of social support perceived by parents of children with CP.

Importantly, the multi-group CFA demonstrated configural, metric, and scalar invariance of the Chinese MSPSS between mothers and fathers of children with CP. This is similar to the results of Cheng and Chan (2004) with the samples of adolescents. To our knowledge, this is the first report of measurement invariance across sex of the Chinese MSPSS in parents of children with CP. This finding indicates that the structure of Chinese MSPSS does measure the same construct for fathers and mothers of children with CP. Thus, in further research the fathers and mothers can be put together without worrying about gender effects on the structure of Chinese MSPSS.

Besides, the Pearson’s correlation analysis revealed that the total MSPSS was significantly related to the total PSI-SF (*r* = –0.313, *p* < 0.01). This is consistent with the previous study on the relationship of MSPSS and PSI-SF for caregivers of children with developmental disabilities (*r* = –0.390, *p* < 0.01) (Peer, 2011). In addition, the three subscales of MSPSS were all significantly related to the three subscales of PSI-SF. The strong correlation between parenting stress and social support confirms that the Chinese version of the MSPSS has good concurrent validity.

### Strengths and Limitations

In summary, the present findings suggest that the Chinese MSPSS displays adequate psychometric properties in terms of internal consistency, factor structure validity, and measurement invariance across gender in parents of children with CP, so it can be applied to these particular populations to measure perceived social support. The availability of the MSPSS might facilitate the examination of the relationship and causal mechanism involved in the link between perceived social support and mental health among Chinese parents of children with CP. Although the present study yielded the expected results, there were still some limitations that should be noted. Firstly, the samples used in the current study are all Chinese, so we cannot be sure that the factor structure and measurement invariance of MSPSS can be obtained in other language versions. Secondly, the study used only cross-sectional data, so we can’t confirm whether our results hold across different time periods.

## Author Contributions

All authors listed have made a substantial, direct and intellectual contribution to the work, and approved it for publication.

## Conflict of Interest Statement

The authors declare that the research was conducted in the absence of any commercial or financial relationships that could be construed as a potential conflict of interest.
